# A Weakly Supervised Learning Method for Cell Detection and Tracking Using Incomplete Initial Annotations

**DOI:** 10.3390/ijms242216028

**Published:** 2023-11-07

**Authors:** Hao Wu, Jovial Niyogisubizo, Keliang Zhao, Jintao Meng, Wenhui Xi, Hongchang Li, Yi Pan, Yanjie Wei

**Affiliations:** 1 Shenzhen Key Laboratory of Intelligent Bioinformatics and Center for High Performance Computing, Shenzhen Institute of Advanced Technology, Chinese Academy of Sciences, Shenzhen 518055, China; wuhaost@mail.ustc.edu.cn (H.W.); jovialniyo93@gmail.com (J.N.); kl.zhao@siat.ac.cn (K.Z.); jt.meng@siat.ac.cn (J.M.); wh.xi@siat.ac.cn (W.X.); 2 University of Chinese Academy of Sciences, Beijing 100049, China; 3 Institute of Biomedicine and Biotechnology, Shenzhen Institute of Advanced Technology, Chinese Academy of Sciences, Shenzhen 518055, China; hc.li@siat.ac.cn; 4 College of Computer Science and Control Engineering, Shenzhen Institute of Advanced Technology, Chinese Academy of Sciences, Shenzhen 518055, China; yi.pan@siat.ac.cn

**Keywords:** cell detection, cell tracking, deep learning, weakly supervised learning, iPS cell reprogramming, brightfield microscopy

## Abstract

The automatic detection of cells in microscopy image sequences is a significant task in biomedical research. However, routine microscopy images with cells, which are taken during the process whereby constant division and differentiation occur, are notoriously difficult to detect due to changes in their appearance and number. Recently, convolutional neural network (CNN)-based methods have made significant progress in cell detection and tracking. However, these approaches require many manually annotated data for fully supervised training, which is time-consuming and often requires professional researchers. To alleviate such tiresome and labor-intensive costs, we propose a novel weakly supervised learning cell detection and tracking framework that trains the deep neural network using incomplete initial labels. Our approach uses incomplete cell markers obtained from fluorescent images for initial training on the Induced Pluripotent Stem (iPS) cell dataset, which is rarely studied for cell detection and tracking. During training, the incomplete initial labels were updated iteratively by combining detection and tracking results to obtain a model with better robustness. Our method was evaluated using two fields of the iPS cell dataset, along with the cell detection accuracy (DET) evaluation metric from the Cell Tracking Challenge (CTC) initiative, and it achieved 0.862 and 0.924 DET, respectively. The transferability of the developed model was tested using the public dataset FluoN2DH-GOWT1, which was taken from CTC; this contains two datasets with reference annotations. We randomly removed parts of the annotations in each labeled data to simulate the initial annotations on the public dataset. After training the model on the two datasets, with labels that comprise 10% cell markers, the DET improved from 0.130 to 0.903 and 0.116 to 0.877. When trained with labels that comprise 60% cell markers, the performance was better than the model trained using the supervised learning method. This outcome indicates that the model’s performance improved as the quality of the labels used for training increased.

## 1. Introduction

### 1.1. Background

Time-lapse microscopy is essential for studying cell proliferation, migration, and other dynamic cellular processes. It helps to understand fundamental biological mechanisms, such as tissue formation and repair, wound healing, and tumor therapy [1]. Detecting and tracking cellular behavior is crucial when studying the biological characteristics of many cells, especially during the early embryonic stages [2]. However, it is difficult to extract and locate the iPS progenitor cells, namely, iPS-forming cells, during the early reprogramming stage; this is because no known biomarkers are available to map iPS progenitor cells [3]. However, after six days of induction, iPS cells can be tested with fluorescent probes, and the proportion of iPS progenitor cells in the early stage of reprogramming is less than 5%. This indicates that the number of iPS cells available during the experimental determination stage is deficient.

Different imaging modalities, including magnetic resonance imaging, mammography, breast sonography, and magnetic resonance tomography, have found extensive applications in medical diagnostics, particularly in histopathology studies [4]. Mammograms, which are valued for their cost-effective high sensitivity, are the preferred method for early detection; they excel in detecting masses and microcalcifications, while maintaining reliability through reduced radiation exposure, thereby contributing significantly to histopathology studies [5]. Recently, a novel framework utilizing neural network concepts and reduced feature vectors, combined with ensemble learning, was introduced, achieving superior accuracy in classifying mitotic and non-mitotic cells in breast cancer histology images, thereby contributing to enhanced cancer diagnoses [6]. Moreover, Labrada and Barkana introduced a breast cancer diagnosis methodology using histopathology images, achieving robust classification via three feature sets and four machine learning (ML) algorithms [7]. Chowanda investigated optimal deep learning (DL) parameters for breast cancer classifications; they introduced a modified architecture and evaluated ML algorithms using mammogram images [8].

Recently, several studies on iPS cell identification during the early stages of reprogramming focused on the analysis of tracking and detection results, as well as the characteristics of cell migration trajectories, so that researchers can understand the characteristics of iPS cells [9,10]. However, modern imaging techniques have created large volumes of data that cannot be analyzed manually. Additionally, it is challenging for specialized personnel to detect low-resolution objects. Traditional cell detection and tracking methods need to design feature extraction operators to capture the unique pattern of each cell type, which usually requires professional knowledge and complex adjustment processes [11]. As presented in natural image tracking benchmarks, traditional methods have dominated cell detection and tracking due to the need for more high-quality annotations [12,13].

Cell detection methods can be classified into three major categories. The first category consists of thresholding methods that separate the cell’s foreground and background in different pixel value ranges [14]. The second category consists of feature extraction methods based on extraction operators, such as the scale-invariant feature transform, followed by a self-labeling algorithm, and two clustering steps to detect unstained cells [15]. The third category consists of edge detection methods that use watersheds to detect cell edges based on gradient changes and corresponding connected regions [16]. A review of cell detection methods for label-free contrast microscopy is provided in Ref. [17].

The two most frequently used approaches for cell tracking are tracking via model evolution and tracking via detection; each approach tackles some challenges more efficiently than the other [18]. Tracking via model evolution method combines segmentation and tracking; this is achieved by describing cell contours over time using an evolving mathematical representation [19]. Despite the ability to produce more accurate contours and individual cell tracks in some applications, these approaches require the initial seeding of cell contours. They could be more computationally efficient for multicell tracking. As examples, parametric models, including an active contour-based “snakes” model in two dimensions [20], and a dynamic mesh or a deformable model in three dimensions [21], may be used. Implicit methods such as the advanced level-set-based multicell segmentation and tracking algorithm and the Chan–Vese Model, may also be used; these methods naturally manage splits, and they merge the new appearances of the cells [22,23].

In tracking by detection, cells are first detected using image segmentation and then tracked to establish correspondence between cells across all frames [19]. Segmentation procedures can be performed via gradient features, intensity features, wavelet decomposition, and region-based or edge-based features [24]. Comparing cell tracking algorithms revealed that most tracking approaches use nearest neighbors, graph-based linking, or multiple hypotheses [13]. The Viterbi algorithm was proposed to link cell outlines generated by a segmentation method into tracks [25]. In Ref. [26], a joint segmentation-tracking algorithm was presented in which the model parameters are learned using Bayesian risk minimization. A comprehensive review of computational methods utilized in cell tracking is provided in Ref. [27].

Artificial intelligence (AI)-/DL-based detection and tracking approaches have made significant progress in this area and have demonstrated better performance when compared to traditional methods [28]. Despite the success of DL methods in tracking multiple objects on natural images, researchers have developed only a few DL approaches for tracking individual cells. Payer et al. [29] presented a method that simultaneously segments and tracks cells using a pixel-wise metric embedding learning strategy combined with a recurrent hourglass network. In Ref. [30], the authors combined a CNN-based observation algorithm with a particle-filter-based method to track non-rigid and non-significant cells.

CNN-based models have demonstrated the ability to perform better in many computer vision tasks without manual annotations [31]. They can work well with prior knowledge on specific tasks to achieve almost the same performance as models trained with manually labeled data [32]. Nishimura et al. proposed weakly supervised cell tracking, which uses the detection results (i.e., the coordinates of cell positions) to train the CNN model without association information, in which nuclear staining can be used to quickly determine cell positions [33]. Similarly, a semi-automated cell tracker was developed using a CNN-based ML algorithm to detect cell nuclei [34]. The detection results were linked at different time points using a minimum-cost flow solver to form cell trajectories [35].

Moreover, a DL technique called cross-modality inference or transformation accurately predicted fluorescent labels from transmitted-light microscopy images of unlabeled biological samples using the training data composed of a pair of images from different imaging modalities [36]. The authors in Ref. [37] revealed that the training dataset formed by a couple of brightfield and fluorescence image modalities of the same cells could be used to train the robust network. This network can learn to predict fluorescent labels from electron microscope images and alleviate the need to acquire the corresponding fluorescence images [37]. In addition, this capability is especially suitable for long-term live-cell imaging, where low phototoxicity acquisitions offer significant benefits. The same method performed better using a small dataset of around 30 to 40 images [28] and could differentiate cell type and subcellular structures [38].

However, state-of-the-art traditional and DL cell tracking methods still rely on the supervision of manual annotations, which is a time-consuming task requiring qualified professional experimenters’ participation [39]. In particular, cell detection and tracking require further development for low signal-to-noise ratio and three-dimensional data [13]. Moreover, the cells may adhere to each other due to persistent cell migration, making it challenging to determine cell boundaries. Similarly, cell division and differentiation processes may lead to continuous changes in appearance, making it difficult to label the cells precisely. Since iPS cells are cultured in a nutrient solution, there are complex texture features in the background of the cell image and the phototoxicity of fluorescence, which can lead to a significant decline in imaging performance. Furthermore, brightfield images are sometimes taken at high magnification, which causes some impurities and bubbles in the images and consequently causes difficulties in cell labeling because experimenters are required to correct repeated errors.

To address these issues, we propose a novel weakly supervised learning method utilizing incomplete initial annotations for automated cell detection and tracking on the iPS cell dataset including brightfield and fluorescence images in the early reprogramming stage. Specifically, we aim to investigate whether this approach can successfully handle the complexities posed by dynamic changes in cell morphology, shape, and thickness during processes such as cell division and differentiation, ultimately achieving performance comparable to or exceeding traditional fully supervised methods. By integrating morphological shape and thickness analysis into the training process, the proposed method demonstrated robust performance, even with incomplete initial annotations, and outperformed traditional fully supervised methods that rely on extensive manual annotations. Inspired by automated curriculum learning [40], the training process starts with incomplete initial annotations generated from paired red fluorescent images taken under the point light. This is similar to beginning training with simple samples in the early stages of curriculum learning. Although all cells cannot be labeled at this stage, we train the model iteratively, where the analysis of cell tracking results is used to update optimized labels in the subsequent training process. Similar to the continuous addition of complex samples in curriculum learning, the updated results are used to continue the training process in our method. Therefore, we updated and improved the incomplete initial labels using the tracking-by-detection algorithm to develop a robust cell detector. This study contributes to advancing the automated microscopy image analysis field, with potential implications for a wide range of biomedical research applications. The code and data for our work, along with more technical details, can be found by visiting the following link: https://github.com/jovialniyo93/cell-detection-and-tracking (accessed on 21 June 2023).

The contributions and novelties of this paper can be summarized as follows:

- To the best of our knowledge, this pioneering study successfully utilizes incomplete initial annotations to develop a robust and universal weakly supervised learning method for automated cell detection and tracking on a brand-new iPS cell reprogramming dataset. The experimental results demonstrate that this study’s developed method achieves a justifiable performance (0.862 and 0.924 DET on two fields of iPS) comparable to the state-of-the-art supervised learning approaches.
- We propose a procedure to obtain incomplete initial labels by removing the possible point light interference from paired red fluorescent images. On other microscopy image datasets, incomplete initial annotations can be obtained using different unsupervised cell detectors such as the Gaussian filter, Cellbow magnification, and automatic thresholding methods provided in ImageJ (version 2.1.0, open source software available at https://github.com/imagej/imagej (accessed on 21 June 2023)), and bilateral filter with prior knowledge of cell shape.
- We demonstrate that our method achieves competitive detection and tracking performance on the public dataset FluoN2DH-GOWT1 from CTC, which contains two datasets with reference annotations. To simulate the incomplete initial annotations, we randomly removed 10%, 20%, 30%, 40%, 50%, 60%, 70%, 80%, and 90% of the cell markers to train the model. The prediction performance on all metrics using partial point annotation is comparable to the model trained using the conventional supervised learning method.
- Since there are few relevant evaluation indicators in cell detection and tracking, we referred to CTC to select the appropriate evaluation metrics to assess the proposed method’s prediction performance.

### 1.2. Literature Review

The precise detection and tracking of cells in microscopy images play a crucial role in various biological tasks [39]. Multiple techniques have been introduced for cell detection and tracking. Recently, the prevailing methods follow a two-step approach: Firstly, they detect cells within each frame, and subsequently, they establish connections between the detected cells across consecutive frames [19]. Various methods have been suggested for detecting cell regions within individual frames, including graph cuts and deep learning [41,42]. The process of associating cells is tackled through linear programming optimization [43]. Alternatively, graph-based optimization techniques have been introduced, wherein an entire sequence of images is represented as a graph, with cells as nodes and association hypotheses as edges, thereby solving for comprehensive solutions [44]. However, these methods rely on the proximity of cell positions for association scores, rendering them ineffective at low frame rates when cells exhibit significant movement.

Recent advancements involve data-driven approaches that employ deep neural networks to estimate cell motion or optical flow. He et al. [30] evaluated motion attributes such as cell motion direction, enlargement, and shape alteration. Nonetheless, this method falters when dealing with densely populated cell scenarios, as it is designed for single-object tracking. Various CNN-based methods for estimating optical flow, like FlowNet [45], have been introduced. Yet, challenges persist in establishing accurate ground-truth data for flow in cell images, and even when estimated, the application of flow in tracking multiple cells within dense environments remains unclear.

The application of AI and DL techniques has shown remarkable progress across various medical domains. Ryu et al. presented SegR-Net, a DL model that combines multiscale feature fusion, deep feature magnification, and precise interference for retinal vessel segmentation [46]. Their framework outperforms existing models in accuracy and sensitivity, and it holds promise for enhancing retinal disease diagnosis and clinical decision-making. Introducing an efficient DL technique, Mangj et al. [47] utilized a multiscale DCNN to detect brain tumors, with a remarkable 97% accuracy on MRI scans of meningioma and glioma cases, demonstrating superiority over prior ML and DL models for tumor classification.

In Ref. [48], the authors introduced RAAGR2-Net, an encoder–decoder-based brain tumor segmentation network leveraging a residual spatial pyramid pooling module and attention gate module to enhance accuracy on multimodal MRI images, surpassing existing methods on the BraTS benchmark. Attallah and Zaghlool [49] introduced a pioneering AI-based pipeline that merges textural analysis and DL to enhance the precision of categorizing pediatric medulloblastoma subtypes from histopathological images. This innovative approach holds significant promise for advancing individualized therapeutic strategies and risk assessment in pediatric brain tumors, underscoring the transformative potential of AI in medical diagnosis and treatment [49].

Recently, U-Net and its variants have become well known, particularly for medical imaging, revolutionizing the way we diagnose and treat. Their innovative architecture empowers precise analysis, aiding doctors’ expertise. With exceptional detection and segmentation capabilities, they unravel intricate details, enhancing our understanding of conditions. A novel modified U-Net architecture was introduced for the accurate and automatic segmentation of dermoscopic skin lesions, incorporating feature map dimension modifications and increased kernels for precise nodule extraction [50].

Similarly, a modified U-Net was introduced to precisely segment diabetic retinopathy lesions, utilizing residual networks and sub-pixel convolution [51]. Rehman et al. [52] proposed BrainSeg-Net, an encoder–decoder model with a feature enhancer block, enhancing spatial detail retention for accurate MR brain tumor segmentation. A UAV-based weed density evaluation method utilizing a modified U-Net is presented, facilitating precise field management [53]. Furthermore, a modified U-Net architecture known as BU-Net was introduced, enhancing accurate brain tumor segmentation through residual extended skip and wide context modifications, along with a customized loss function, thereby improving feature diversity and contextual information and enhancing segmentation performance [54].

In this study, we introduce an innovative, weakly supervised learning method designed for detecting and tracking cells, leveraging incomplete initial annotations. This approach adds to the current spectrum of AI models and directly confronts the labor-intensive demands inherent in conventional, fully supervised training. Building upon these advancements, our work extends the research frontier by presenting a novel strategy that effectively harnesses the potential of incomplete initial labels for training purposes. This strategic enhancement significantly alleviates the burden of extensive manual annotations, thus enabling a more streamlined and efficient approach to developing accurate cell detection and tracking models.

## 2. Results

### 2.1. Results of Processing Fluorescent Images

**Figure 1:**
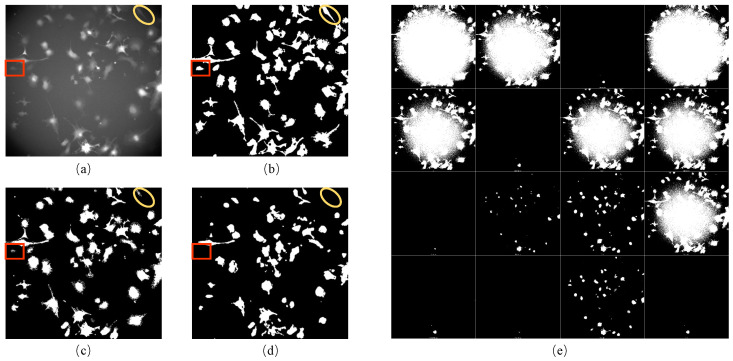
Results of processing fluorescent images: (**a**) the red fluorescent image; (**b**) the result of (**a**) after processing using our method: CLAHE + bilateral filter; (**c**) the result of (**a**) after processing using the Gaussian filter; (**d**) the result of (**a**) after processing using Cellbow magnification; (**e**) the result of (**a**) after processing using the 16 auto-threshold methods provided in ImageJ (version 2.1.0). The red boxes and yellow circles show the cell masks around the boundary of the fluorescent image, which can be processed correctly using our method only.

In this study, we developed a unique method to generate incomplete initial labels of brightfield images using paired red fluorescent images. The technique combines CLAHE and bilateral filter to binarize the fluorescent images correctly (Figure 1b). We used this approach because the fluorescent images were taken under the point light. Moreover, our method was compared with the existing competitive benchmark approaches that can be used on other microscopy image datasets when brightfield and red fluorescence images are unavailable. As the pixel value in the middle area of the fluorescent image was more significant than in the edge, the 16 automatic thresholding methods provided in ImageJ software (version 2.1.0) were not able to correctly binarize the red fluorescence images in our iPS cell dataset (Figure 1e). The pixel value distribution was determined by the distribution of point light intensity, which was very close to the Gaussian distribution. Therefore, the conventional Gaussian filter method was used to remove uneven illumination. However, this method was unsuitable for our dataset’s fluorescent images (Figure 1c). To confirm the effectiveness of our method, we applied a DL method known as “Cellbow” which is commonly used to process any fluorescent image [55]. Still, the result after using this method was unsatisfactory for our data (Figure 1d).

### 2.2. Improvement in Pseudo-Ground Truth in the Training Process

In the proposed method, the pseudo-ground truth processed from fluorescent images was used for initialization to obtain a well-trained cell detector. Without manual annotation, we trained model M1 on F1 and model M2 on F2 datasets. These two models were then used to detect the cells on each other’s dataset. We took manually annotated data as GT during the testing and initialized them as the baseline. All the metrics were calculated in comparison to GT. To analyze the effect of tracking, we ran our methods on three iPS early reprogramming period datasets. Specifically, we used weighted sum with and without tracking analysis to update pseudo-ground truth for the next round of training. The pseudo-ground-truth performance results, before and after training with our method, are shown in Table 1 and Table 2.

Compared with the pseudo-ground-truth performance, the *DET* improved by 0.185, 0.111, and 0.141 for the model trained with tracks in the three periods of the F1 dataset. Moreover, the performance results improved by 0.119, 0.079, and 0.091 when the model was trained without tracks. In comparison with the effect of tracking analysis, the results show that our method performed better when trained with tracks than when trained without tracks in terms of *DET*. However, the reverse scenario was observed regarding precision and *N_fp_*/image. The reason is that tracking analysis removed FP errors when updating labels for the next round of training. Therefore, the label noise was reduced, enabling the model to learn from more correct features and predict more positives in the next iteration. Due to the limitations of tracking analysis, not all FP errors were removed. This resulted in poor performance in precision and *N_fp_*/image metrics. Since tracking analysis in our method helped the model to predict more positives, the *N_fn_*/image was less. While calculating the *DET*, different errors were assigned different weights by considering the cost of correcting them. The weight of FP errors was 1, while the weight of FN errors was 10, indicating that the *DET* achieved a better score for fewer FN errors. Although our method with track performed worse on precision and *N_fp_*/image, the performance of this method was deemed better than that method trained without tracks.

We computed the *TRA* to assess the effectiveness of the tracking algorithm. Since our method relied on analyzing the tracking results, the effectiveness of tracking determined the accuracy of removing FP errors in detection results. Compared with the calculation of *DET, TRA* considered three more errors: redundant edges to be deleted, with weight 1; edges to be added, with weight 1.5; and edges with wrong semantics to be deleted, with weight 1. Since the weights of these errors are small, the value of *TRA* is always smaller than *DET*. As the values of *TRA* in Table 1 are smaller than *DET* values, and the difference is not significant, we considered the tracking algorithm based on the overlap as applicable. The effectiveness of the tracking algorithm was assessed on the F2 dataset. From Table 2, it can be inferred that the *TRA* has the same tendency as in Table 1. Therefore, our method performed better when trained with tracks than without tracks on all evaluation metrics except precision and *N_fp_*/image.

**Figure 2:**
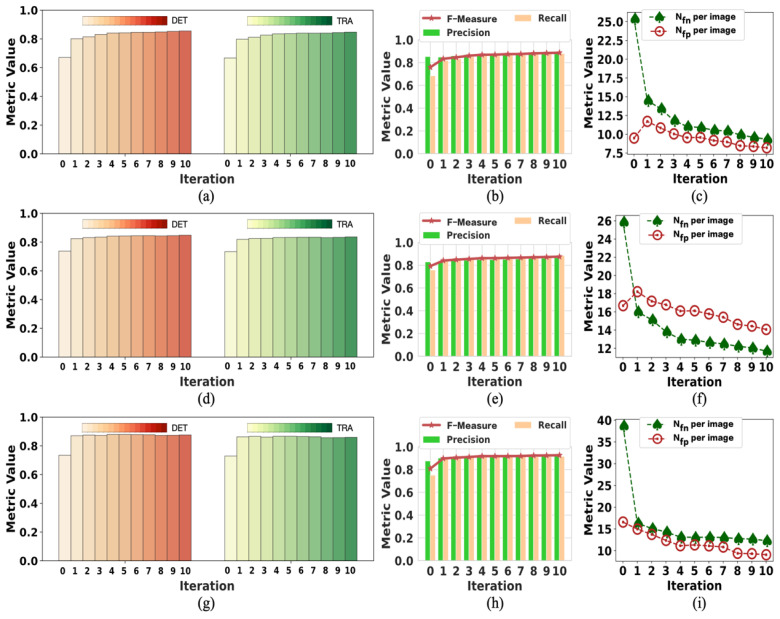
The change in initial labels on F1 during the training process. Iteration 0 represents the initialization; the data are the same as the “initial” in Table 1. The data of iteration 10 are the same as “with tracks” in Table 1: (**a**–**c**) are the performance results in period 1; (**d**–**f**) are the performance results in period 2; (**g**–**i**) are the performance results in period 3.

**Figure 3:**
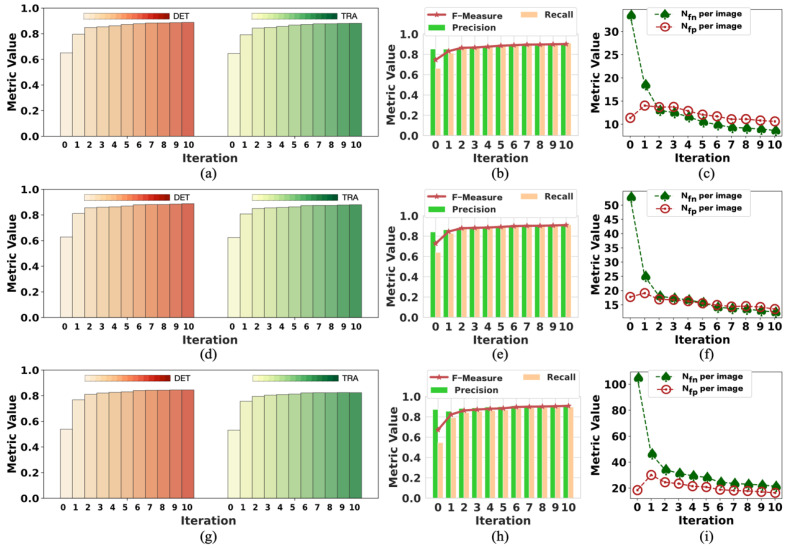
The change in initial labels on F2 during the training process. Iteration 0 represents the initialization; the data are the same as the “initial” in Table 2. The data of iteration 10 are the same as “with tracks” in Table 2: (**a**–**c**) are the performance results in period 1; (**d**–**f**) are the performance results in period 2; (**g**–**i**) are the performance results in period 3.

Figure 2 presents the change in labels on F1 in each training iteration using our method with tracking analysis, while Figure 3 shows the performance on F2. The initial label is denoted as iteration 0, showing the changes from iteration 1 to 10. The data of iteration 0 in Figure 2 and Figure 3 are the same data of initial labels provided in Table 1 and Table 2. The improvement in the quality of labels is observed over the iterations. As shown in Figure 2a,d,g, iteration 1 achieved a significant *DET* increase. The model’s performance on *DET* and *TRA* changed a little after iteration 5, but the value of *N_fn_* per image and *N_fp_* per image kept dropping in the subsequent few iterations. In iteration 1, the value of *N_fp_* per image increased because of the failure to determine and remove a moderate amount of FP errors. After iteration 5, the value of *N_fp_* per image was lower than the initial value. Because of the difficulty of detecting iPS cells in the early reprogramming period, the changing tendency of evaluation metrics over iterations was different but overall consistent. Thus, the quality of labels at iterations did not show any degradation due to the combined operation of weighted sum with pseudo-ground truth.

### 2.3. Cross-Dataset Performance Evaluation on Our Data

We applied the models trained to detect iPS cells on F1 and F2 datasets. The model trained on F1 was M1, while the model trained on F2 was M2. Model M1 was tested on the F2 dataset, while model M2 was tested on F1. The models’ performances tested with and without tracking were compared to study the effect of tracking analysis. Table 3 shows the performance of model M1 tested with and without tracking, while Table 4 presents the performance of model M2 tested with and without tracking.

As shown in Table 3 and Table 4, with the results highlighted in bold, the model tested with tracking achieved better scores than the model tested without tracking for all evaluation metrics. The results indicate that tracking analysis in our method significantly improved the model’s performance because, after removing the FP errors in labels with the help of tracking analysis, the model was not affected by noise. As was expected, M2 performed better and achieved the average *DET* value of 0.924 in the three periods because M2 was trained with complex samples but tested with simple ones (as the cell density of F2 was greater than that of F1). However, the scenario was the opposite for M1. Under this circumstance, M1 still achieved the average *DET* value of 0.862 in the three periods.

**Figure 4:**
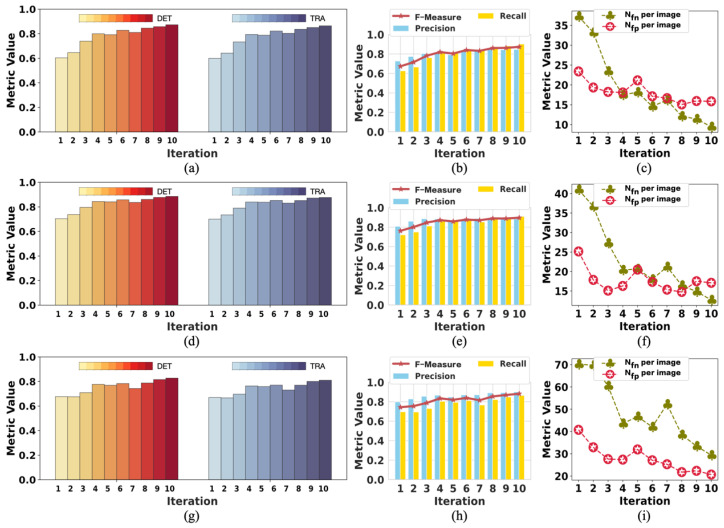
The change in the performance of M1 when testing on F2: (**a**–**c**) are the performance results in period 1; (**d**–**f**) are the performance results in period 2l (**g**–**i**) are the performance results in period 3. The model achieved the best performance in iteration 10 on all the metrics.

**Figure 5:**
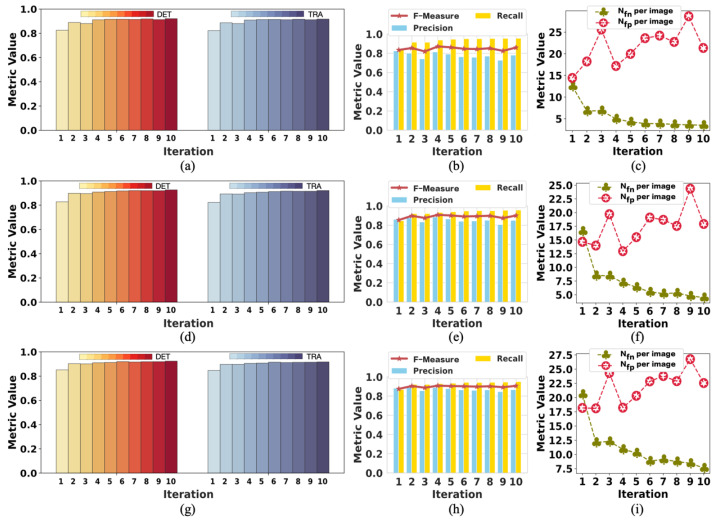
The change in the performance of M2 when testing on F1: (**a**–**c**) are the performance results in period 1; (**d**–**f**) are the performance results in period 2; (**g**–**i**) are the performance results in period 3. The model performed best in iteration 10 on almost all the metrics except *N_fp_* per image.

To analyze the changes in model performance during the testing process, we checked the model’s performance after each iteration. The performance results of the model tested with tracking analysis are summarized in Figure 4 and Figure 5, respectively. The results of iteration 10 in these two figures are the same data provided in Table 3 and Table 4, respectively. Figure 4 shows the performance changes in M1, while Figure 5 shows the changes in M2 during the testing process.

In Figure 4, the trend of rising volatility of evaluation metrics can be observed. This is partly related to the model being tested on complex samples. Moreover, we controlled the number of times the network updates the parameters in each iteration by less than 100 to reduce the noise during the training process. This also reduced the ability of the model to learn from the labels in the current iteration. Therefore, the model’s performance decreased even when the labels were improved compared with the last iteration. As shown in Figure 4, the performance results fluctuated, but the evaluation metrics showed a trend of improved model performance.

Because M2 was tested on simple samples, there was a slight fluctuation in the evaluation metrics, as shown in Figure 5. It was easier for M2 to obtain good performance with minor changes in the evaluation results. However, the *N_fp_* per image could have been better because the data (F1) used to test M2 contained more light-spot noise with the same appearance as cells. Because the light-spot noise was not present in the training data (F2) of M2, M2 mistakenly took the light-spot noise as cells. Nevertheless, the *N_fn_* per image was relatively low. Therefore, we considered M2 to perform well. Both models achieved the best performance on iteration 10, affirming the positive effect of updating labels on model performance during training. This also confirmed the effectiveness of our weakly supervised method designed for cell detection.

### 2.4. Performance on the Fluo-N2DH-GOWT1 Dataset

#### 2.4.1. The Effect of Initial Labels’ Quality Improvement in the Training Process

To prove the effectiveness of our method, we tested it on a public dataset by studying the effect of the initial labels’ quality improvement on our approach. The transferability of the developed method was tested on the Fluo-N2DH-GOWT1 cell dataset from CTC. We chose the Fluo-N2DH-GOWT1 dataset to test our approach because it has brightfield images with strong contrast. Fluo-N2DH-GOWT1 contains two training datasets with reference annotations. We followed the same procedure on the iPS cell dataset to test the developed method on these two training datasets. The same evaluation metrics were used to assess the performance of our approach on these public datasets. We randomly removed 10%, 20%, 30%, 40%, 50%, 60%, 70%, 80%, and 90% of the cell markers in each labeled label to simulate the pseudo-ground truth used to train the model. The two datasets are denoted as S1 and S2. After the training process, the improvement in initial labels for S1 and S2 are shown in Table 5 and Table 6, respectively. The models trained on S1 and S2 are denoted as U1 and U2, respectively.

To demonstrate the performance of refining the labels of our method during training, we used different percentages of labels (*L*_per_) to start training. The 10% *L*_per_ in Table 5 and Table 6 means that we randomly removed 90% of the annotations in each label before training, which was later updated 10 times during the training process. The evaluation metrics of the updated labels were calculated after 10 iterations. As shown in Table 5 and Table 6, the *DET* and *Recall* values for the initial labels are very close to the proportion of cell markers used while training the model. The *N_fn_*/image values gradually decrease as the proportion of cell markers used for model training increases. Additionally, it can be observed that the *N_fn_*/image values of S2 are greater than those of S1, reflecting that the cell density of S1 is smaller than that of S2. However, the cells may adhere to each other regardless of the density, making it challenging to determine cell boundaries accurately.

Although the training started from 10% of labels, the labels were updated precisely, and consequently, the *DET* value was updated from 0.130 to 0.903 (Table 5). With an improvement in the quality of initial labels, the refined labels show a trend of improvement after training. When the percentage of annotations used for training was 90%, the updated labels achieved the *DET* value of 0.981. Without any doubt, the developed method effectively refined the initial labels during the training process. When more than 60% of annotations were used for training, the *DET* values of the final labels after 10 iterations changed with a slight difference (0.1). The results in Table 6 show the same trend as those in Table 5.

#### 2.4.2. Performance of Cell Detection Method Using Labels with Different Qualities and Cross-Dataset Performance Evaluation

This section explores the impact of training with different initial label annotations on model performance. The models obtained in the previous section were used to test the performance on opposite datasets. Moreover, the performance of these models was compared with the model trained with the fully supervised learning method using different evaluation indicators. Table 7 presents the cell detection performance of model U1 trained on S1 with different initial labels and tested on S2 except for the last row. The table’s last row shows the model’s performance trained with the normal supervised learning method. The training epochs were set to 30, and the batch size was set to 8 using the same input and DL network. The last row illustrates the best model performance after 30 training epochs. The results on other rows were calculated using a model trained for 10 iterations with our method.

As shown in Table 7, even when the model was trained with 100% of reference annotations using the normal supervised learning method, the model performance was not better than the model trained with 60% of reference annotations using our method. The reason is that the model trained with our approach gained more robustness during training. In the updating process of the labels, like in curriculum learning, simple samples were used at the beginning, and complex samples were used during the next training process. It was found that the FN errors in the labels functioned like regularization to prevent the model from overfitting issues. After training the model with the same iterations, we observed an improvement in model performance for our method.

Even when training with 10% of annotations, the model achieved the acceptable *DET* value of 0.849. When 90% of annotations were used for training, the model trained with our method achieved a *DET* value of 0.967, significantly better than the model trained with full supervision. Table 8 shows the cell detection performance of model U2 trained on S2 with different initial labels and tested on S1 except for the last row. The results in Table 8 have the same trend as those in Table 7. As can be observed, the performance of U2 on S1 is better than that of U1 on S2, which aligns with the expected outcomes.

#### 2.4.3. Performance of Cell Detection Method by Replacing U-Net with Another Deep Learning Architecture

We performed an evaluation study to show that the methodology proposed in this study works when U-Net is replaced by other advanced DL architectures. On the Fluo-N2DH-GOWT1 method, we replaced U-Net with a CTC algorithm named “MU-US” in our dataset. The MU-US algorithm was proposed with a custom deep convolutional U-Net named “U-SE-ResNet”. U-SE-ResNet is an encoder–decoder architecture with a ResNet-50 backbone used for feature extraction in the encoder module [56]. The overall architecture of this model is similar to U-Net. The encoder part of this method consists of residual blocks equipped with squeeze and excitation blocks and skip connections after each block of ResNet-50 [56]. During the training process for this method, we used the same hyperparameters and training configurations used to train U-Net.

In this evaluation study, the S1 and S2 datasets of Fluo-N2DH-GOWT1 were used to train the proposed models denoted as D1 and D2, respectively. For testing these models, the performance of D1 was tested on S2, while the performance of D2 was tested on S1. Following the procedure described in Section 2.4.2, we compared the model’s performance trained with different initial annotations with a fully supervised learning method. Table 9 shows the performance of model D1 trained on S1 with different initial labels and tested on S2. The results obtained using the model trained with U-SE-ResNet show the same trend as those obtained using the model trained with U-Net, although there is a slight difference. The model trained with 60% of reference annotations performed better than that trained with 100% of reference annotations using the normal supervised learning method. We used tracking-assisted correction to update the model iteratively. Table 10 shows the cell detection performance of model D2 trained on S2 with different initial labels and tested on S1. The results in Table 10 have the same trend as those in Table 9. Our method is recommended or uniformly justifiable based on the results of replacing U-Net with U-SE-ResNet. The weakly supervised learning method proposed in this study can work efficiently using different DL architectures.

Due to the unique nature of our microscope and microscopy data, which have never been used in similar research, it is difficult to compare our results with those of other researchers. Despite this, the performance of the proposed method in this study was compared with the CTC state-of-the-art cell detection and tracking algorithms from different participants on a public dataset. It should be noted that in CTC, all datasets in every category were used for training. As mentioned in the previous sections, we performed the training on one dataset and tested it on the other. On the Fluo-N2DH-GOWT1 dataset used to evaluate our method, the best-performing algorithm, namely “TUG-AT”, proposed cosine embeddings combined with recurrent fully convolutional hourglass networks to segment and track cell instances [29]. The second method, named “KTH-SE”, used a tracking-by-detection architecture that relied on four separate segmentation algorithms and a Viterbi-based track-linking method. The third method, named “BGU-IL”, used a hybrid of convolutional long short-term memory and U-Net to incorporate temporal information that can facilitate the detection and tracking of individual touching cells or partially visible cells [57]. As shown in Table 11, our method can be ranked second in terms of *TRA* and first in terms of *DET*.

## 3. Materials and Methods

**Figure 6:**
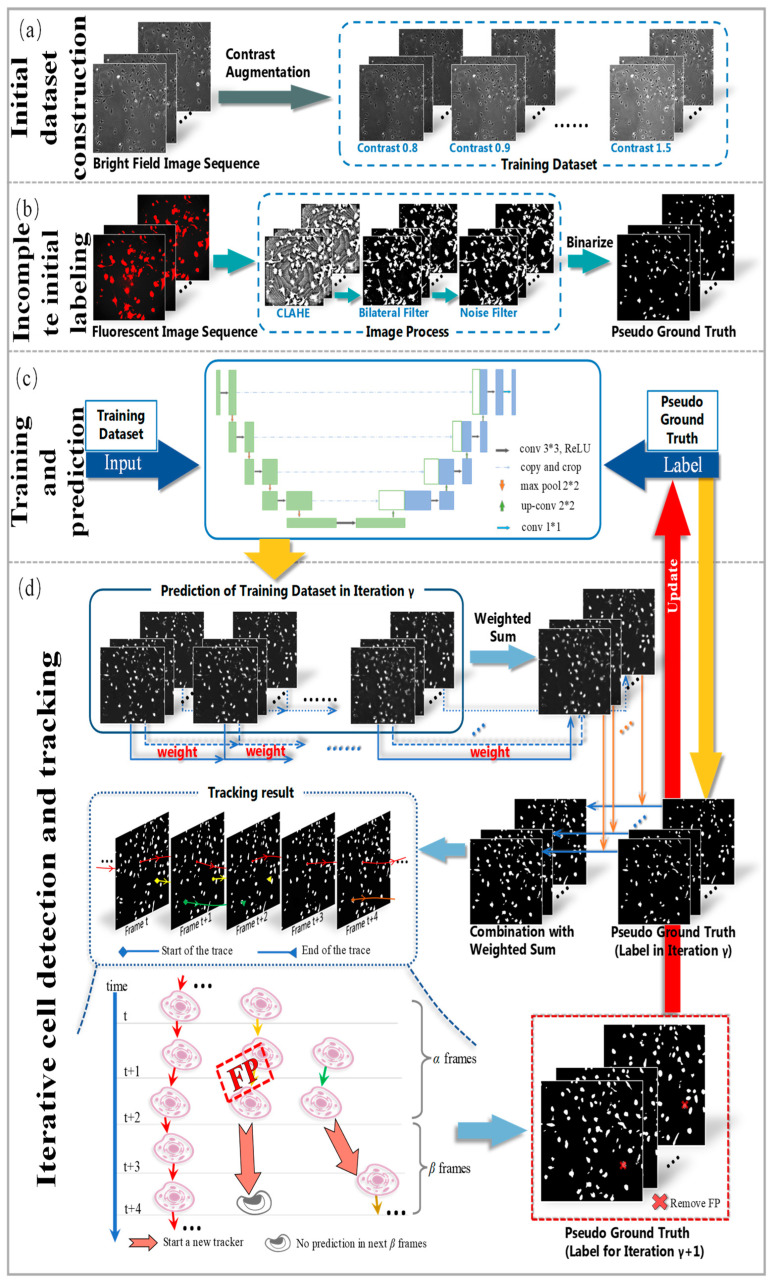
The framework of the proposed method. From top to bottom are initial dataset construction, incomplete initial labeling, the training and prediction process, and iterative cell detection and tracking.

The overall workflow of the proposed method is presented in Figure 6. It mainly includes the initial dataset construction, the incomplete initial labeling, the training and prediction process, and the iterative cell detection and tracking procedure. Firstly, brightfield cell images were taken as the input of the CNN model. Moreover, contrast augmentation was applied to expand the training dataset (Figure 6a). Then, by processing fluorescent cell images, incomplete initial annotations of the nucleus were generated using the contrast limited adaptive histogram equalization (CLAHE) and bilateral filter methods. These incomplete annotations/labels of cells were treated as initial pseudo-ground truth (Figure 6b). In the next stage, the U-Net model was used for cell detection on the dataset with the initial pseudo-ground truth as initial labels (Figure 6c). In the final step (Figure 6d), the training process was divided into several iterations, during which the U-Net model predicted the cells in all frames. We analyzed the tracking results by conducting cell tracking on the predictions and removing the incorrect masks in the forecast. The pseudo-ground truth was updated in this way for the next iteration. The details of the developed approach are described in the following sections.

### 3.1. Live Cell Imaging of iPS Cell Reprogramming

The cell images were generated using an Olympus IX81 live cell imaging system equipped with a 10× UPlanFL objective iXon3 EMCCD camera sourced from Tokyo, Japan. iPS cell reprogramming was performed for mouse embryonic fibroblasts (MEFs). A detailed description of cell culture and the generation of iPS cells can be found in Ref. [58]. From Day 0, the images of mouse embryonic fibroblasts (MEFs) were taken for 135 h and 40 min. For the first 48 h and 40 min, brightfield and red fluorescence images were acquired at an interval of 10 min. After two days of dual-channel imaging, a green fluorescence channel was added to indicate the expression of GFP-Oct4, and the acquisition interval was adjusted to 30 min. Motorized stage control was used to track cells in the same field, and 33 fields were selected each time for further analysis. Cell images taken within the first 48 h and 40 min since Day 0 were used to construct the dataset because, after this time, the GFP-Oct4 was added to identify the progenitor cells experimentally. Overall, 233 brightfield and the same number of red fluorescence images for the dataset at each field correspond to the first three periods.

### 3.2. Dataset Construction and Independent Test Dataset

#### 3.2.1. Data Augmentation for Brightfield Cell Images

Data augmentation operations help gain the model’s robustness by increasing the training dataset. Regular data augmentation operations for images include changing contrast, brightness, zooming, rotating, mirror filling, cropping, and elastic deformation. These operations can be combined with different parameters. Because of uneven luminous intensity distribution produced by the point light source, cells in various fields and periods show different intensities. In this paper, we applied contract augmentation for the brightfield microscopic images. By changing the contrast of the brightfield images, more input samples for the DL model were generated to reduce overfitting and obtain a more robust prediction model. The protocol to change the contrast of brightfield images was to multiply the pixel value by a parameter determined manually by the next steps.

Firstly, in the training dataset, the original brightfield and augmented images were combined. Moreover, the training dataset with the partially labeled data was used to train the U-Net model. If the number of markers in the predicted results was more significant than that in the partial labels, we selected this parameter since the change in contrast by multiplying this parameter would help the model to learn more characteristics and gain robustness. After a few tests, we set the parameters for changing contrast to values varying from 0.8 to 1.5 with an interval of 0.1. The parameters resulted in 8 times larger datasets (F1 and F2). Since both the F1 and F2 datasets contained 233 brightfield images, the total images for both datasets increased to 1864 after data augmentation. For the Fluo-N2DH-GOWT1 dataset, the same data augmentation technique was used.

#### 3.2.2. Incomplete Initial Labeling of Brightfield Images Using the Paired Red Fluorescent Images

**Figure 7:**
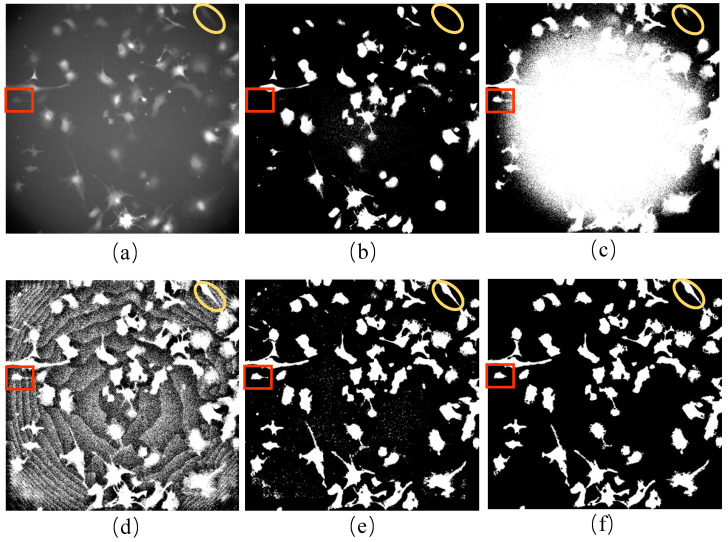
Initial labeling of brightfield images: (**a**) red fluorescent image of the first frame for the F1 dataset; (**b**) the binarization of (**a**) with a threshold of 23; (**c**) the binarization of (**a**) with a threshold of 16; (**d**) the fluorescent image processed using CLAHE; (**e**) the result of (**d**) processed using the bilateral filter; (**f**) the result after noise removal in (**e**). Images in (**e**,**f**) are binarized for visualization. (Red squares and yellow ellipsis represent the sample of cells taken for initial labeling).

Incomplete initial labels represent the pseudo-ground truth for the brightfield images. The incomplete initial labels were generated from the paired red fluorescent images taken under the point light. The variation in light source intensity affects the quality of an image, and the pixel values in the central area of red fluorescent images are usually higher than those around the cells. This complicates the process of image binarization. It is believed that increasing the threshold value makes cell masks more transparent when the number of cells is comparatively small. Figure 7 shows the first frame of the red fluorescent images of the F1 dataset (Figure 7a), together with the results using different binarization techniques (Figure 7b–f). A threshold value of 23 for binarization made the cell masks around the boundary area challenging to detect (Figure 7b). In contrast, a threshold value of 16 made it difficult to distinguish between the cell masks in the central region and those from the background (Figure 7c).

The red fluorescent images were processed in four steps. Firstly, CLAHE was performed to equalize the pixel value (Figure 7d). In the second step, a bilateral filter was applied to reduce the noise and protect the edge of cell masks at the same time (Figure 7e). Thirdly, noises with a smaller area than cells in the images were removed by comparing the size of cell markers (Figure 7f). Finally, thresholding was used to binarize the red fluorescent images as incomplete initial labels. Instead of using the automated binarization method in the last step, we determined the threshold manually to detect the nucleus better and track the cells simultaneously. Overall, we generated the initial labels of the cells in the brightfield images using the paired red fluorescent images. However, because not all cells could be transfected successfully, only cells showing the red fluorescence signals were labeled to obtain the incomplete initial labels considered pseudo-ground truth.

#### 3.2.3. Ground-Truth Labels of Brightfield Cell Images

Among all the 33 field images, 2 fields with different cell densities were selected to make manual annotations used as ground truth (GT) to evaluate the performance of our method. The dataset with a small cell density was denoted as F1, while the dataset with a large cell density was denoted as F2. Both datasets were used to train the proposed algorithms denoted as M1 and M2, respectively. For testing the M1 and M2 models, the performance of M1 was tested on F2, while the performance of M2 was tested on F1. We developed a program based on OpenCV and red fluorescent image processing to annotate the cells manually in brightfield images. The brightfield image was superimposed on the paired red fluorescent image with the help of an interactive window, and the paired red fluorescent image was used as a reference for cell labeling.

The left mouse button is used to draw a mask on the fluorescent image, while the right mouse button is used to erase the mask. The program allows for using a stylus pen for more precise cell labeling. After manual labeling, cell tracking tasks were performed on the annotated cells based on the overlap method to refine the annotations. To ensure that the cells were labeled correctly, more than two researchers carefully evaluated the tracking results frame by frame to remove the wrong annotations or add the missing ones. This step was repeated until the researchers found no errors. In this paper, two researchers performed the entire process, and the cell annotations obtained with consensus were taken as the GT. In the ground truth, cell multi-division and cell fusion events were excluded since they represent a deficient number of annotations.

#### 3.2.4. Independent Test Datasets

The transferability of the proposed model was tested on the Fluo-N2DH-GOWT1 dataset from CTC. Leica TCS SP5 equipped with a Plan-Apochromat 63×/1.4 (oil) objective lens was used to investigate whether Oct4 recognized damaged chromatin in mouse ESCs expressing GFP-Oct4. The images were taken at an interval of 5 min. The details of this dataset can be found in Ref. [59]. The training dataset with reference annotations was used for evaluation. The training data were divided between two sub-datasets, denoted in this paper as S1 and S2. Both of them had 92 brightfield images with reference annotations. The S1 and S2 datasets were used to train the proposed algorithms/models denoted as U1 and U2, respectively. For testing the U1 and U2 models, the performance of U1 was tested on S2, while the performance of U2 was tested on S1.

### 3.3. Training and Prediction

#### 3.3.1. Cell Detection for Brightfield Images Using Incomplete Annotations

The essence of weakly supervised learning is DL, with the deep neural network as the core. Cell detection is a binary classification task where the cells to be detected are regarded as the foreground and background as a class. The label value corresponding to the cell is 1, while the label value corresponding to the background is 0. U-Net has garnered recognition for its exceptional performance in addressing intricate image segmentation tasks. We decided to use U-Net as the cell detector in our method because it has achieved excellent ranking on many ISBI Cell Tracking Challenge datasets [60,61,62]. However, many binary classification models such as ResNet [63], fully convolutional neural networks [64], Mask RCNN [65], and other advanced DL architectures can be used as cell detectors for subsequent training processes. This study extends the conventional approach and introduces a custom deep convolutional U-Net called “U-SE-ResNet” as a replacement for U-Net to prove the effectiveness and adaptability of the proposed methodology [56].

Initially created for cell segmentation, U-Net proves its mettle in achieving superior performance with a relatively small volume of training data. As shown in Figure 6b, the contraction part (left side, encoder) of the proposed U-Net in our method contains four blocks and skip connections after each block. Each block consists of two 3 × 3 convolutions and a downsampling max pooling operation. The first layer of the contraction part consists of the input layer, which accepts gray images with the size 736 × 736. After each downsampling process, the length and width of the feature map are divided into two. The expansion part of the network (right side, decoder) also consists of four blocks, each containing one deconvolution and two convolution operations. The deconvolution operation doubles the length and width of the feature map. Then, it merges with the feature map obtained in the contraction path at the same level for the successive two convolution operations. Due to the symmetry of the encoder and decoder structures, the model generates the exact size of the corresponding marker as the original image. Our model had 31,402,501 trainable parameters with a computation time of 3 h.

The computation time of 3 h was achieved during the training process of our weakly supervised learning method. The experiments were conducted on a robust computing system featuring four NVIDIA Tesla P100 GPUs. These GPUs, known for their parallel processing capabilities, played a pivotal role in accelerating the training process. The system operated on a CentOS Linux release 7.4.1708 operating system, with computational tasks managed with an Intel Xeon E5-2650 v4 CPU boasting 128 GB of RAM, sourced and operated in Santa Clara, CA, USA. While specific details about processor speeds and other hardware specifications are not provided, this carefully configured setup undoubtedly contributed to the attainment of the stated computation time. The training process was implemented using Python version 3.8 within a PyTorch DL framework, highlighting the utilization of cutting-edge tools to achieve efficient and accurate results.

After thoroughly searching for the best hyperparameters and training configurations, we used the binary cross-entropy loss function and Adam optimizer parameters to train the model. The learning rate was set to 0.0001. We divided the whole training process into 10 iterations. The pseudo-ground truth of the brightfield images was updated and improved iteratively. We trained our algorithm on two datasets of iPS cell reprogramming denoted as F1 and F2, respectively. Each dataset has 1864 augmented images. The batch size was set to 24, implying that the model was updated 78 times for each epoch. The two models trained on F1 and F2 datasets were denoted as M1 and M2, respectively. The performance of M1 was tested on F2, while the performance of M2 was tested on F1. For the Fluo-N2DH-GOWT1 dataset, we trained our methods on the S1 and S2 datasets. Both datasets contained 92 images, and the total number of images increased to 736 after data augmentation. The batch size was set to 8, implying that the model was updated 92 times for each epoch. The two models trained on S1 and S2 were denoted as U1 and U2, respectively. The performance of S1 was tested on U2, while the performance of S2 was tested on U1.

#### 3.3.2. Prediction of Cell Masks Using the Weighted Sum for the Training Dataset

**Figure 8:**
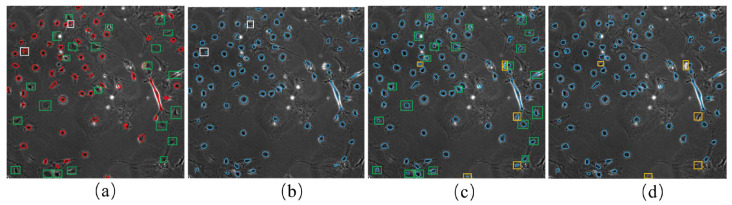
The prediction of cell masks by weighted sum: (**a**) shows the initial pseudo-ground truth of the first frame of F1. The red circles indicate cell marks of the initial pseudo-ground truth; (**b**) cell masks from the weighted sum of the eight different predictions (blue circles); (**c**) a combination of cell masks from weighted sum prediction (**b**) and the initial pseudo-ground truth (**a**); (**d**) shows the final cell masks after applying the tracking-assisted correction to the cell masks in (**c**). Green boxes in (**a**,**c**) indicate the FN errors in pseudo-ground truth (**a**), which were predicted correctly in (**c**). White boxes in (**a**,**b**) indicate the cells for which the model predicted worse results than the pseudo-ground truth. Yellow boxes in (**c**,**d**) show the FP errors in the weighted sum, which were determined and removed using the tracking-assisted correction.

During each iteration, the model predicted eight results for the brightfield image corresponding to eight additional augmented images. To enhance the reliability of the prediction results, we calculated the weighted sum of the eight outcomes. The exact weight of 0.125 was used for all eight different results. For example, Figure 8a shows the pseudo-ground truth of the first frame of the F1 dataset. The predicted cell masks from the weighted sum on the same image are demonstrated in Figure 8b in blue circles. The weighted sum prediction and the pseudo-ground truth were combined, as shown in Figure 8c. Inevitably, the model sometimes showed poor performance due to its strong ability to fit the FN errors in the pseudo-ground truth, indicated by the green boxes in Figure 8a,c. In other cases, some cells were not predicted even when they had the corresponding masks in pseudo-ground truth, as shown by the white boxes in Figure 8a,b.

When comparing the incomplete labels with the weighted sum results, it is clear that the latter can annotate more cells. However, some cells with the corresponding annotations could not be detected with the weighted sum. Thus, when using the weighted sum results to replace the incomplete labels to continue training, different FP errors were introduced while reducing some FN errors. We used the union of the initial labels and the weighted sum results to address this issue. For the convenience of the following description, we refer to this union as the cell detection results. The cell detection results contained some incorrect annotations (FP errors), which were reduced in subsequent analyses using cell tracking.

### 3.4. Iterative Cell Detection and Tracking

#### 3.4.1. Tracking by Detection

Given the cell detection results for all time-lapse brightfield images, the cell tracking problem is to find the successor in frame *t + 1* for the cell in frame *t*. We used the intersection between adjacent cell masks to track cells. Assuming the area of cell masks between intersections as $A_{t}$ and $A_{t+1}$ in frame *t* and *t + 1*, respectively, the overlap is calculated as follows:

(1) $$Overlap=\;\frac{A_{t}\cap^{\;}A_{t+1}}{A_{t}}$$

We detected the nucleus area, and changing its location as the cell moved was easy. When finding the candidate cells in frame *t + 1*, we dilated the cell mask in frame *t*. We chose the one where the overlap exceeded the threshold to determine the specific cell successor. This strategy was also applicable to the cell division scenarios. The threshold value depends on several factors such as the time interval between the frames, cell speed, and cell area. Generally, cell tracking based on overlap worked well when the threshold value was set to 0.1. Even if the markers belonging to the same cell in two adjacent frames could not be associated due to some unreasonable setting of this threshold, they would be associated in the subsequent analysis of tracking results.

#### 3.4.2. Tracking-Assisted Correction

In the brightfield images, some impurities and bubbles similar to cells under high magnification appeared, which made it difficult for the model to distinguish them. This resulted in incorrect annotations (FP errors) in the cell detection results. We believe that these FP errors shared the same features, with shorter trajectories than cells. For our dataset, we set α to 3 and β to 5, which stated that if no prediction was associated in the subsequent 5 frames, the mask with a trajectory length shorter than 3 was determined as FP and removed from the pseudo-ground truth. We set α to 3 and β to 8 for the Fluo-N2DH-GOWT1 dataset. We analyzed the tracking results and started a new tracker for the markers whose length of trajectories was shorter than α. FP errors were determined and removed from the pseudo-ground truth. When choosing the FP, the following two factors were taken into account:

Location: The cells around the boundary of the brightfield image entered and left the image field frequently, making their trajectory relatively short and hard to associate. From the trajectory perspective analysis, these cells were treated as noise. Their removal had no impact on model performance. Therefore, the cells near the border of the image were not analyzed. Although some noise might be missed, this had no significant effects on cell detection prediction performance.

The acceptable maximum number of frames for missing markers: For the possible FP, we started a new tracker to check if there was any prediction associated with the subsequent β frames. To find the candidate cells in the successive β frames, the area of the mask in the last trajectory frame was dilated three times. In addition, the overlap with the subsequent β frames was calculated to obtain the candidate cells. Once the cell mask with no parents appearing in the next β frames had the overlap exceeding the threshold of 0.1, this cell was taken as the successor.

### 3.5. Evaluation Metrics

In this paper, we used the following evaluation metrics to measure the performance of the proposed method:

**DET:** This is an evaluation metric for detection accuracy used in CTC [13]. *DET* evaluates the detection accuracy by comparing the nodes of acyclic-oriented graphs representing objects in both the GT and the detection results from the tested method. Numerically, *DET* is a normalized acyclic oriented graph matching (*AOGM-D*) measure for detection and is defined as follows [66]:

(2) $$DET=1-\frac{\min\left(AOGM-D,AOGM-D_{0}\right)}{AOGM-D_{0}}$$

where AOGM−D is the cost of transforming a set of nodes predicted using the tested method into the set of GT nodes; $AOGM-D_{0}$ denotes the cost of creating the set of GT nodes from scratch; and AOGM indicates empty detection results. The *DET* value always falls in the [0,1] interval, with higher values corresponding to better detection performance [13].

**TRA:** This is also an evaluation metric used in CTC. It evaluates the tracking accuracy of the tested method measured by comparing the tracked objects with the gold standard reference annotation of selected frames. Numerically, *TRA* is a normalized acyclic oriented graph matching (*AOGM*) measure defined as follows [66]:

(3) $$TRA=1-\frac{\min\left(AOGM,AOGM_{0}\right)}{AOGM_{0}}$$

where $AOGM_{0}$ is the cost required for creating the reference graph from scratch, and AOGM is the value for empty tracking results. The *TRA* value always falls in the [0,1] interval, with higher values corresponding to better tracking performance [13].

**Precision:** This metric is used to evaluate the percentage of true-positive instances in the overall positive instances of objects in the detector based on the reference annotation. It is defined as follows:

(4) $$Precision=\frac{TP}{TP+FP}$$

**Recall:** This metric is used to evaluate the ratio of true positives that have been predicted in the ground truth, defined as follows:

(5) $$Recall=\frac{TP}{TP+FN}$$

**F-Measure:** The harmonic mean of *Precision* and *Recall* is defined as follows:

(6) $$F-Measure=\frac{2\times Precision\times Recall}{Precision+Recall}$$

***N_fn_*/*image*:** This metric measures the average number of cell markers not given in every image. It is defined as follows:

(7) $$N_{fn}/image=\frac{FN}{num\;of\;image}$$

***N_fp_*/*image*:** This metric measures the average number of wrong cell markers in the prediction results per image. It is defined as follows:

(8) $$N_{fp}/image=\frac{FP}{num\;of\;image}$$

## 4. Conclusions

In this paper, we proposed a novel weakly supervised learning method for cell detection and tracking using incomplete initial annotations in brightfield microscopy images. Using the tracking-by-detection algorithm, we updated the incomplete initial labels to train a robust cell detector. The whole process was automated without using manually labeled data. We proposed a procedure to remove the point light effect in fluorescent images to obtain incomplete initial labels. On other microscopy image datasets different from ours, incomplete initial labels can be obtained using some unsupervised cell detectors with prior knowledge of cell shape, while our procedure relies on fluorescent images. Once the initial labels are acquired, our method can be used to train a robust cell detector. After cell detection, tracking through detection can be efficiently conducted. Our method can analyze large volumes of data with comparable performance as fully supervised methods while requiring less annotation work.

The proposed method was extensively evaluated on two fields of brand-new iPS cell reprogramming datasets. Its competitive performance was confirmed on the public FluoN2DH-GOWT1 from CTC, which contains two datasets with reference annotations. The detection and tracking power of the developed model was evaluated using two appropriate evaluation metrics from CTC (*DET* and *TRA*). The average *DET* values for three periods achieved on the two fields of the iPS dataset were 0.862 and 0.924, while the *TRA* values were 0.850 and 0.918. We simulated the incomplete initial annotations on open data with reference annotations by randomly removing 10%, 20%, 30%, 40%, 50%, 60%, 70%, 80%, and 90% of the cell markers to train the model. Moreover, the performance was evaluated using the fully supervised learning method. On FluoN2DH-GOWT1, the *DET* improved from 0.130 to 0.903 and 0.116 to 0.877, while the *TRA* improved from 0.115 to 0.899 and 0.102 to 0.874 after training the model on the two datasets with labels containing only 10% of cell markers. After training the model with 60% of cell markers, the *DET* improved from 0.616 to 0.978 and 0.610 to 0.990, and the *TRA* improved from 0.586 to 0.972 and 0.579 to 0.989.

Although our method uses only weak labels, the updated performance results after training the model with 60% of cell markers prove that our method outperforms many state-of-the-art fully supervised learning methods. Using a normal supervised learning method on FluoN2DH-GOWT1 data, the *DET* values obtained were 0.938 and 0.974, while the TRA values were 0.938 and 0.970. The outcomes of this paper can help to know the trajectories of cells and function as a reference for manual annotations. While the method demonstrates promising performance on specific datasets, some limitations warrant consideration. Notably, the framework’s effectiveness is contingent upon the quality of initial annotations and the accuracy of preprocessing steps, which could impact its generalization to diverse microscopy contexts.

Furthermore, while the reduction in annotation effort is advantageous, the potential trade-off between effort and performance should be carefully evaluated. Future research endeavors could explore advancements in the method’s robustness, broader applicability, and possible extension to other deep learning models to address these concerns. By openly acknowledging these limitations and emphasizing the method’s unique contributions, we aim to engage in constructive discourse and advance the field of weakly supervised cell analysis. Furthermore, we plan to study the effect of applying test-time/prediction-time augmentation and the impact of using limited artificially labeled datasets to learn how cell density, shape, and texture affect the model’s performance.

### Implications of the Study

The implications of this study extend to both the scientific and practical realms of cell analysis. Scientifically, our proposed weakly supervised framework challenges the conventional reliance on extensive manual annotations, paving the way for more efficient and scalable methods for studying cell dynamics. By demonstrating competitive performance on diverse datasets, the study highlights the potential of utilizing incomplete initial labels to gain insights into cellular behaviors, ultimately aiding researchers in understanding complex biological processes.

From a practical standpoint, the developed framework is promising as it significantly reduces labor-intensive annotation efforts in cell detection and tracking tasks. This efficiency gain can accelerate research timelines and make large-scale analyses more accessible to a broader range of researchers, fostering advances in various fields of biomedicine and beyond. Moreover, the framework’s adaptability to different datasets and potential integration with more advanced deep learning models underscores its potential for broader applications in real-world scenarios, ranging from drug discovery to disease modeling.

## Figures and Tables

**Table 1:** The improvement in the quality of labels on the F1 dataset after training with our method. Performance results with and without tracking analysis are compared.

Label	With Track?	Period	*DET*	*TRA*	*Precision*	*Recall*	*F-Measure*	*N_fn_*/Image	*N_fp_*/Image
Initial		1	0.670761	0.667061	0.852820	0.684108	0.759204	25.4	9.5
		2	0.737573	0.733189	0.829384	0.758094	0.792138	25.9	16.7
		3	0.734766	0.729094	0.875594	0.750769	0.808391	38.7	16.6
Updated	no	1	0.797005	0.791304	**0.901262**	0.812964	0.854839	14.8	**7.1**
		2	0.816979	0.806617	**0.872666**	0.846190	0.859224	15.9	**12.8**
		3	0.825262	0.813154	0.903727	0.854198	0.878265	21.8	13.6
Updated	yes	1	**0.855386**	**0.847222**	0.893354	**0.880216**	**0.886736**	**9.3**	8.2
		2	**0.848170**	**0.836255**	0.865533	**0.885551**	**0.875427**	**11.7**	14.1
		3	**0.875933**	**0.859624**	**0.936484**	**0.915652**	**0.925943**	**12.3**	**9.0**

“Initial” means the initial labels obtained from red fluorescent images used to start the training. “Updated” means the initial labels are updated after the whole training process with our method. The results highlighted in bold represent better performance.

**Table 2:** The improvement in the quality of labels on the F2 dataset after training with our method. The performance results with and without tracking analysis are compared.

Label	With Track?	Period	*DET*	*TRA*	*Precision*	*Recall*	*F-Measure*	*N_fn_*/Image	*N_fp_*/Image
Initial		1	0.650393	0.645579	0.852676	0.662388	0.745582	33.5	11.4
		2	0.627827	0.624200	0.840979	0.639854	0.726758	52.9	17.8
		3	0.538057	0.531580	0.872758	0.546432	0.672077	105.0	18.4
Updated	no	1	0.854522	0.847030	**0.926850**	0.871536	0.898342	12.5	**6.7**
		2	0.806824	0.801072	**0.928571**	0.819106	0.870410	26.0	**9.1**
		3	0.661730	0.646834	**0.923722**	0.679573	0.783059	69.8	**12.2**
Updated	yes	1	**0.888517**	**0.881664**	0.892712	**0.910498**	**0.901517**	**8.7**	10.6
		2	**0.888062**	**0.880135**	0.905251	**0.912114**	**0.908670**	**12.4**	13.5
		3	**0.845088**	**0.823904**	0.919397	**0.896841**	**0.907979**	**21.4**	16.3

The results highlighted in bold represent better performance.

**Table 3:** Comparison of the performance of M1 when testing on F2. The performance of the model with and without tracking analysis is compared.

Model	With Track?	Period	*DET*	*TRA*	*Precision*	*Recall*	*F-Measure*	*N_fn_*/Image	*N_fp_*/Image
M1	no	1	0.849872	0.844170	0.802526	0.877614	0.838392	12.0	21.2
		2	0.873369	0.868922	0.855620	0.894630	0.874690	15.2	21.8
		3	0.799094	0.786991	0.865893	0.825839	0.845392	39.0	28.6
M1	yes	1	**0.873171**	**0.864446**	**0.845831**	**0.902042**	**0.873033**	**9.4**	**15.8**
		2	**0.885290**	**0.876665**	**0.883186**	**0.910496**	**0.896633**	**12.7**	**17.1**
		3	**0.827788**	**0.810312**	**0.900545**	**0.864268**	**0.882034**	**29.4**	**20.6**

The results highlighted in bold represent better performance.

**Table 4:** The performance of M2 when testing on F1. The performance of the model with and without tracking analysis is compared.

Model	With Track?	Period	*DET*	*TRA*	*Precision*	*Recall*	*F-Measure*	*N_fn_*/Image	*N_fp_*/Image
M2	no	1	0.906027	0.904693	0.749562	0.943057	0.835249	4.6	25.2
		2	0.894712	0.889356	0.825507	0.926369	0.873035	7.8	20.8
		3	0.858742	0.852928	0.850220	0.882034	0.865835	18.4	24.2
M2	yes	1	**0.920833**	**0.917558**	**0.780920**	**0.955900**	**0.859596**	**3.5**	**21.3**
		2	**0.926640**	**0.921108**	**0.849632**	**0.957574**	**0.900379**	**4.5**	**17.9**
		3	**0.923739**	**0.917361**	**0.866850**	**0.950723**	**0.906851**	**7.6**	**22.6**

The results highlighted in bold represent better performance.

**Table 5:** The improvement in the quality of labels on S1 after training with our method. To test the performance, we used difficult-quality initial labels for training.

*L*_per_	State	*DET*	*TRA*	*Precision*	*Recall*	*F-Measure*	*N_fn_*/Image	*N_fp_*/Image
10%	initial	0.130224	0.115625	0.992537	0.129377	0.228916	19.5	0.0
	updated	0.903499	0.899080	0.899420	0.920792	0.909980	1.7	2.3
20%	initial	0.219631	0.196848	0.995575	0.218872	0.358852	17.5	0.0
	updated	0.926385	0.916323	0.900529	0.959058	0.928872	0.9	2.3
30%	initial	0.323372	0.295377	0.996992	0.322628	0.487500	15.1	0.0
	updated	0.962391	0.954258	0.941771	0.983912	0.962380	0.3	1.3
40%	initial	0.423955	0.393272	0.997706	0.423358	0.594465	12.8	0.0
	updated	0.965355	0.956924	0.893713	0.991968	0.940281	0.2	2.6
50%	initial	0.513362	0.481752	0.998108	0.512895	0.677596	10.9	0.0
	updated	0.963946	0.959568	0.871166	0.989055	0.926375	0.2	3.2
60%	initial	0.616861	0.586671	0.998424	0.616545	0.762335	8.6	0.0
	updated	0.978814	0.972813	0.993497	0.992504	0.993000	0.2	0.1
70%	initial	0.721331	0.694594	0.998652	0.721168	0.837525	6.2	0.0
	updated	0.983090	0.978123	0.959350	0.998507	0.978537	0.0	0.9
80%	initial	0.824830	0.805797	0.998821	0.824818	0.903518	3.9	0.0
	updated	0.986054	0.980895	0.980976	0.997520	0.989179	0.1	0.4
90%	initial	0.914237	0.902994	0.998937	0.914355	0.954776	1.0	0.0
	updated	0.981147	0.973744	0.987562	0.998993	0.993245	0.0	0.3

*L*_per_: percentage of annotations in each label used for training. 10% means only 10% of annotations in each label were used for training.

**Table 6:** The improvement in the quality of the labels on S2 after training with our method. To test the performance, we used difficult-quality initial labels for training.

*L*_per_	State	*DE* *T*	*TRA*	*Precision*	*Recall*	*F-Measure*	*N_fn_*/Image	*N_fp_*/Image
10%	initial	0.116011	0.102517	0.993151	0.115308	0.206626	24.2	0.0
	updated	0.877116	0.874489	0.909873	0.887690	0.898645	3.1	2.4
20%	initial	0.211760	0.190179	0.996248	0.211133	0.348425	21.6	0.0
	updated	0.961661	0.960489	0.957724	0.967665	0.962669	0.9	1.2
30%	initial	0.316647	0.289650	0.997491	0.316103	0.480072	18.7	0.0
	updated	0.9631303	0.958861	0.906273	0.977053	0.940333	0.6	2.7
40%	initial	0.412396	0.380913	0.998073	0.411928	0.583169	16.1	0.0
	updated	0.973023	0.970774	0.964131	0.980361	0.972178	0.5	1.0
50%	initial	0.509337	0.477024	0.998440	0.508946	0.674216	13.4	0.0
	updated	0.983830	0.982080	0.990381	0.986821	0.988598	0.4	0.3
60%	initial	0.610648	0.579109	0.998699	0.610338	0.757651	10.7	0.0
	updated	0.990266	0.989438	0.987322	0.992038	0.989674	0.2	0.3
70%	initial	0.719507	0.693244	0.998896	0.719284	0.836338	7.7	0.0
	updated	0.993643	0.992901	0.972050	0.997212	0.984470	0.1	0.8
80%	initial	0.824394	0.804921	0.999036	0.824254	0.903268	4.8	0.0
	updated	0.992809	0.991429	0.988920	0.994033	0.991470	0.2	0.3
90%	initial	0.920540	0.910312	0.999137	0.920477	0.958195	2.2	0.0
	updated	0.997735	0.997022	0.992490	0.998410	0.995441	0.0	0.2

*L*_per_: percentage of annotations in each label used for training. 10% means only 10% of annotations in each label were used for training.

**Table 7:** The performance of U1 when testing on S2. The performance of the model trained with different initial labels is compared, with 100% *L*_per_ indicating the model trained with full reference annotations like the normal supervised learning method, which uses the same U-Net within our method but skips the updating procedure.

*L*_per_	*DET*	*TRA*	*Precision*	*Recall*	*F-Measure*	*N_fn_*/Image	*N_fp_*/Image
10%	0.849352	0.845280	0.949266	0.855939	0.900190	3.9	1.2
20%	0.843226	0.834753	0.954984	0.861583	0.905882	3.6	1.1
30%	0.849821	0.846180	0.921484	0.862333	0.890928	3.7	1.9
40%	0.867104	0.863114	0.934792	0.877921	0.905464	3.3	1.7
50%	0.892531	0.890591	0.919625	0.903407	0.911444	2.6	2.1
60%	0.942114	0.939521	0.944577	0.952553	0.948549	1.3	1.5
70%	0.954350	0.951468	0.942700	0.964659	0.953553	1.0	1.6
80%	0.957211	0.955831	0.927475	0.967962	0.947286	0.9	2.0
90%	0.966706	0.964454	0.962361	0.974719	0.968501	0.7	1.0
100%	0.938657	0.938275	0.961071	0.942346	0.951616	1.6	1.0

The model trained using 100% of reference annotations was set to 30 epochs, and the best performance was achieved on epoch 21.

**Table 8:** The performance of U2 when testing on S1. The performance of the model trained with different initial labels is compared.

*L*_per_	*DET*	*TRA*	*Precision*	*Recall*	*F-Measure*	*N_fn_*/Image	*N_fp_*/Image
10%	0.904665	0.902740	0.882824	0.920059	0.901057	1.8	2.7
20%	0.947036	0.942516	0.897649	0.967694	0.931356	0.7	2.4
30%	0.963703	0.958997	0.884168	0.990481	0.934310	0.2	2.8
40%	0.970019	0.966466	0.917587	0.987611	0.951313	0.3	2.0
50%	0.973372	0.968878	0.909008	0.994030	0.949620	0.1	2.2
60%	0.976336	0.971522	0.917928	0.996020	0.955381	0.1	1.9
70%	0.976531	0.972072	0.909173	0.998504	0.951747	0.0	2.2
80%	0.976579	0.974019	0.914247	0.991146	0.951145	0.2	2.9
90%	0.980855	0.978314	0.939070	0.992138	0.964875	0.2	1.4
100%	0.974150	0.970951	0.913934	0.990133	0.950509	0.2	2.1

Here, 100% *L*_per_ means the model trained with full reference annotations like the normal supervised learning method, which uses the same U-Net within our method but skips the updating procedure. The model trained using 100% labels was set to 30 epochs, and the best performance was achieved on epoch 20.

**Table 9:** The performance of D1 when testing on S2. The performance of the model trained with different initial labels is compared, with 100% *L*_per_ indicating the model trained with full reference annotations like the normal supervised learning method, which uses the same U-SE-ResNet within our method but skips the updating procedure.

*L*_per_	*DET*	*TRA*	*Precision*	*Recall*	*F-Measure*	*N_fn_*/Image	*N_fp_*/Image
10%	0.808701	0.808349	0.935130	0.814240	0.870508	5.0	1.5
20%	0.825904	0.823118	0.939530	0.833133	0.883138	4.5	1.4
30%	0.855185	0.854578	0.926306	0.863418	0.893757	3.7	1.8
40%	0.936631	0.935141	0.960553	0.941410	0.950885	1.5	1.0
50%	0.932261	0.929393	0.960262	0.939478	0.949756	1.6	1.0
60%	0.947477	0.946482	0.945167	0.953062	0.949098	1.2	1.5
70%	0.953159	0.951659	0.977189	0.956539	0.966753	1.1	0.6
80%	0.956099	0.955433	0.939076	0.962226	0.950510	1.0	1.7
90%	0.952523	0.951295	0.984577	0.982279	0.948549	0.4	1.6
100%	0.942034	0.941997	0.940433	0.947912	0.944158	1.4	0.9

The model trained using 100% of reference annotations was set to 30 epochs.

**Table 10:** The performance of D2 when testing on S1. The performance of the model trained with different initial labels is compared, with 100% *L*_per_ indicating the model trained with full reference annotations like the normal supervised learning method, which uses the same U-SE-ResNet within our method but skips the updating procedure.

*L*_per_	*DET*	*TRA*	*Precision*	*Recall*	*F-Measure*	*N_fn_*/Image	*N_fp_*/Image
10%	0.937707	0.931979	0.895473	0.962387	0.927725	0.8	2.4
20%	0.948008	0.943764	0.921455	0.973094	0.919708	0.5	3.1
30%	0.949077	0.945012	0.930688	0.964338	0.947214	0.7	1.5
40%	0.941642	0.926563	0.945674	0.977639	0.961390	0.4	1.1
50%	0.98241	0.979964	0.927164	0.995573	0.960151	0.0	1.7
60%	0.980515	0.9766	0.940102	0.996033	0.967260	0.0	1.3
70%	0.980855	0.977171	0.928077	0.997522	0.961547	0.0	1.6
80%	0.98139	0.978631	0.920382	0.995570	0.956501	0.0	1.9
90%	0.984645	0.981784	0.931891	0.998520	0.964056	0.0	1.6
100%	0.981487	0.978504	0.925332	0.9960552	0.9593920	0.0	0.9

The model trained using 100% of the labels was set to 30 epochs.

**Table 11:** Quantitative results of the ISBI CTC dataset for *DET* and *TRA* as published on the CTC website.

Fluo-N2DH-GOWT1 Dataset		
Ranking	*TRA*	*DET*
1st	0.979 ^(1)^	0.980 ^(1)^
2nd	0.976 ^(2)^	0.977 ^(2)^
3rd	0.967 ^(3)^	0.970 ^(3)^
Ours	0.978	0.980

The number in parentheses represents the ranking on the CTC website (blue: TUG-AT; green: KTH-SE; orange: BGUIL; yellow: UFRGS-BR; purple: ours).

## Data Availability

The original datasets used to evaluate our method were obtained from a publicly available source [13], and further raw data as well as the codes used in this paper can be found by visiting the following link: https://github.com/jovialniyo93/cell-detection-and-tracking (accessed on 21 June 2023).

## Abbreviations

AI	Artificial intelligence
DL	Deep learning
CNN	Convolutional neural network
iPS	Induced pluripotent Stem
CTC	Cell Tracking Challenge
ML	Machine learning
CLAHE	Contrast limited adaptive histogram equalization
MEFs	Mouse embryonic fibroblasts
FP	False positive
GT	Ground truth
AOGM	Acyclic oriented graph matching

## References

[1] Guan T., Li J., Chen C., Liu Y. (2022). Self-assembling peptide-based hydrogels for wound tissue repair. Adv. Sci..

[2] Nasrollahpour H., Khalilzadeh B., Naseri A., Yousefi H., Erk N., Rahbarghazi R. (2022). Electrochemical biosensors for stem cell analysis; applications in diagnostics, differentiation and follow-up. Trends Analyt. Chem..

[3] Geuder J., Wange L.E., Janjic A., Radmer J., Janssen P., Bagnoli J.W., Müller S., Kaul A., Ohnuki M., Enard W. (2021). A non-invasive method to generate induced pluripotent stem cells from primate urine. Sci. Rep..

[4] Mohapatra S., Muduly S., Mohanty S., Ravindra J.V.R., Mohanty S.N. (2022). Evaluation of deep learning models for detecting breast cancer using histopathological mammograms Images. Sustain. Oper. Comput..

[5] Ragab D.A., Sharkas M., Marshall S., Ren J. (2019). Breast cancer detection using deep convolutional neural networks and support vector machines. PeerJ.

[6] Rehman M.U., Akhtar S., Zakwan M., Mahmood M.H. (2022). Novel architecture with selected feature vector for effective classification of mitotic and non-mitotic cells in breast cancer histology images. Biomed. Signal Process. Control..

[7] Labrada A., Barkana B.D. Breast cancer diagnosis from histopathology images using supervised algorithms. Proceedings of the 2022 IEEE 35th International Symposium on Computer-Based Medical Systems (CBMS).

[8] Chowanda A. (2022). Exploring the Best Parameters of Deep Learning for Breast Cancer Classification System. Commit J..

[9] Yan Y., Wu R., Bo Y., Zhang M., Chen Y., Wang X., Huang M., Liu B., Zhang L. (2020). Induced pluripotent stem cells-derived microvesicles accelerate deep second-degree burn wound healing in mice through miR-16-5p-mediated promotion of keratinocytes migration. Theranostics.

[10] Neavin D., Nguyen Q., Daniszewski M.S., Liang H.H., Chiu H.S., Wee Y.K., Senabouth A., Lukowski S.W., Crombie D.E., Lidgerwood G.E. (2021). Single cell eQTL analysis identifies cell type-specific genetic control of gene expression in fibroblasts and reprogrammed induced pluripotent stem cells. Genome Biol..

[11] Molina-Moreno M., González-Díaz I., Sicilia J., Crainiciuc G., Palomino-Segura M., Hidalgo A., Díaz-De-María F. (2022). ACME: Automatic feature extraction for cell migration examination through intravital microscopy imaging. Med. Image Anal..

[12] Dendorfer P., Rezatofighi H., Milan A., Shi J., Cremers D., Reid I., Roth S., Schindler K., Leal-Taixe L. (2019). CVPR19 tracking and detection challenge: How crowded can it get?. arXiv.

[13] Ulman V., Maška M., Magnusson K.E.G., Ronneberger O., Haubold C., Harder N., Matula P., Matula P., Svoboda D., Radojevic M. (2017). An objective comparison of cell-tracking algorithms. Nat. Methods.

[14] Voigt S.P., Ravikumar K., Basu B., Kalidindi S.R. (2021). Automated image processing workflow for morphological analysis of fluorescence microscopy cell images. JOM.

[15] Ghaznavi A., Rychtáriková R., Saberioon M., Štys D. (2022). Cell segmentation from telecentric bright-field transmitted light microscopy images using a Residual Attention U-Net: A case study on HeLa line. Comput. Biol. Med..

[16] Li X., Jiao H., Wang Y. (2020). Edge detection algorithm of cancer image based on deep learning. Bioengineered.

[17] Vicar T., Balvan J., Jaros J., Jug F., Kolar R., Masarik M., Gumulec J. (2019). Cell segmentation methods for label-free contrast microscopy: Review and comprehensive comparison. BMC Bioinform..

[18] Yi W., Fang Z., Li W., Hoseinnezhad R., Kong L. (2020). Multi-frame track-before-detect algorithm for maneuvering target tracking. IEEE Trans. Veh. Technol..

[19] Wang J., Su X., Zhao L., Zhang J. (2020). Deep reinforcement learning for data association in cell tracking. Front. Bioeng. Biotechnol..

[20] Reddy Soora N., Rahman Mohammed E.U., Waseem Mohammed S., Santosh Kumar N. (2022). Deep Active Contour-Based Capsule Network for Medical Image Segmentation. IETE J. Res..

[21] Dufour A., Thibeaux R., Labruyere E., Guillen N., Olivo-Marin J.-C. (2010). 3-D active meshes: Fast discrete deformable models for cell tracking in 3-D time-lapse microscopy. IEEE Trans. Image Process..

[22] Maška M., Daněk O., Garasa S., Rouzaut A., Muñoz-Barrutia A., Ortiz-De-Solorzano C. (2013). Segmentation and shape tracking of whole fluorescent cells based on the Chan–Vese model. IEEE Trans. Med. Imaging.

[23] Dzyubachyk O., Van Cappellen W.A., Essers J., Niessen W.J., Meijering E. (2010). Advanced level-set-based cell tracking in time-lapse fluorescence microscopy. IEEE Trans. Med. Imaging.

[24] Zebari D.A., Zeebaree D.Q., Abdulazeez A.M., Haron H., Hamed H.N.A. (2020). Improved threshold based and trainable fully automated segmentation for breast cancer boundary and pectoral muscle in mammogram images. IEEE Access.

[25] Magnusson K.E.G., Jalden J., Gilbert P.M., Blau H.M. (2014). Global linking of cell tracks using the Viterbi algorithm. IEEE Trans. Med. Imaging.

[26] Sixta T., Cao J., Seebach J., Schnittler H., Flach B. (2020). Coupling cell detection and tracking by temporal feedback. Mach. Vis. Appl..

[27] Emami N., Sedaei Z., Ferdousi R. (2021). Computerized cell tracking: Current methods, tools and challenges. Vis. Inform..

[28] Caicedo J.C., Goodman A., Karhohs K.W., Cimini B.A., Ackerman J., Haghighi M., Heng C., Becker T., Doan M., McQuin C. (2019). Nucleus segmentation across imaging experiments: The 2018 Data Science Bowl. Nat. Methods.

[29] Payer C., Štern D., Feiner M., Bischof H., Urschler M. (2019). Segmenting and tracking cell instances with cosine embeddings and recurrent hourglass networks. Med. Image Anal..

[30] He T., Mao H., Guo J., Yi Z. (2017). Cell tracking using deep neural networks with multi-task learning. Image Vis. Comput..

[31] Holm E.A., Cohn R., Gao N., Kitahara A.R., Matson T.P., Lei B., Yarasi S.R. (2020). Overview: Computer vision and machine learning for microstructural characterization and analysis. Metall. Mater. Trans. A.

[32] Hossain T., Teng S.W., Sohel F., Lu G. (2021). Robust image classification using a low-pass activation function and dct augmentation. IEEE Access.

[33] Nishimura K., Hayashida J., Wang C., Ker D.F.E., Bise R. (2020). Weakly-supervised cell tracking via backward-and-forward propagation. Proceedings of the Computer Vision–ECCV 2020: 16th European Conference.

[34] Cornwell J., Li J., Mahadevan S., Draper J., Joun G., Zoellner H., Asli N., Harvey R., Nordon R. (2020). TrackPad: Software for semi-automated single-cell tracking and lineage annotation. SoftwareX.

[35] Kok R.N.U., Hebert L., Huelsz-Prince G., Goos Y.J., Zheng X., Bozek K., Stephens G.J., Tans S.J., van Zon J.S. (2020). OrganoidTracker: Efficient cell tracking using machine learning and manual error correction. PLoS ONE.

[36] Meijering E. (2020). A bird’s-eye view of deep learning in bioimage analysis. Comput. Struct. Biotechnol. J..

[37] Von Chamier L., Laine R.F., Henriques R. (2019). Artificial intelligence for microscopy: What you should know. Biochem. Soc. Trans..

[38] Christiansen E.M., Yang S.J., Ando D.M., Javaherian A., Skibinski G., Lipnick S., Mount E., O’Neil A., Shah K., Lee A.K. (2018). In silico labeling: Predicting fluorescent labels in unlabeled images. Cell.

[39] Liu Z., Jin L., Chen J., Fang Q., Ablameyko S., Yin Z., Xu Y. (2021). A survey on applications of deep learning in microscopy image analysis. Comput. Biol. Med..

[40] Graves A., Bellemare M.G., Menick J., Munos R., Kavukcuoglu K. (2017). Automated curriculum learning for neural networks. Int. Conf. Mach. Learn..

[41] Hajdowska K., Student S., Borys D. (2022). Graph based method for cell segmentation and detection in live-cell fluorescence microscope imaging. Biomed. Signal Process. Control..

[42] Jiang H., Diao Z., Shi T., Zhou Y., Wang F., Hu W., Zhu X., Luo S., Tong G., Yao Y.-D. (2023). A review of deep learning-based multiple-lesion recognition from medical images: Classification, detection and segmentation. Comput. Biol. Med..

[43] Kanade T., Yin Z., Bise R., Huh S., Eom S., Sandbothe M.F., Chen M. Cell image analysis: Algorithms, system and applications. Proceedings of the 2011 IEEE Workshop on Applications of Computer Vision (WACV).

[44] Zhang Y., Sheng H., Wu Y., Wang S., Ke W., Xiong Z. (2020). Multiplex labeling graph for near-online tracking in crowded scenes. IEEE Internet Things J..

[45] Dosovitskiy A., Fischer P., Ilg E., Hausser P., Hazirbas C., Golkov V., Van Der Smagt P., Cremers D., Brox T. Flownet: Learning optical flow with convolutional networks. Proceedings of the IEEE International Conference on Computer Vision.

[46] Ryu J., Rehman M.U., Nizami I.F., Chong K.T. (2023). SegR-Net: A deep learning framework with multi-scale feature fusion for robust retinal vessel segmentation. Comput. Biol. Med..

[47] Mangj S.M., Hussan P.H., Shakir W.M.R. (2023). Efficient Deep Learning Approach for Detection of Brain Tumor Disease. Int. J. Online Biomed. Eng. iJOE.

[48] Rehman M.U., Ryu J., Nizami I.F., Chong K.T. (2023). RAAGR2-Net: A brain tumor segmentation network using parallel processing of multiple spatial frames. Comput. Biol. Med..

[49] Attallah O., Zaghlool S. (2022). AI-based pipeline for classifying pediatric medulloblastoma using histopathological and textural images. Life.

[50] Anand V., Gupta S., Koundal D., Nayak S.R., Barsocchi P., Bhoi A.K. (2022). Modified U-net architecture for segmentation of skin lesion. Sensors.

[51] Sambyal N., Saini P., Syal R., Gupta V. (2020). Modified U-Net architecture for semantic segmentation of diabetic retinopathy images. Biocybern. Biomed. Eng..

[52] Rehman M.U., Cho S., Kim J., Chong K.T. (2021). Brainseg-net: Brain tumor mr image segmentation via enhanced encoder–decoder network. Diagnostics.

[53] Zou K., Chen X., Zhang F., Zhou H., Zhang C. (2021). A field weed density evaluation method based on uav imaging and modified u-net. Remote Sens..

[54] Rehman M.U., Cho S., Kim J.H., Chong K.T. (2020). Bu-net: Brain tumor segmentation using modified u-net architecture. Electronics.

[55] Zhang H., Shao X., Peng Y., Teng Y., Saravanan K.M., Zhang H., Li H., Wei Y. (2019). A novel machine learning based approach for iPS progenitor cell identification. PLoS Comput. Biol..

[56] Bártová E., Šustáčková G., Stixová L., Kozubek S., Legartová S., Foltánková V. (2011). Recruitment of Oct4 protein to UV-damaged chromatin in embryonic stem cells. PLoS ONE.

[57] Loffler K., Mikut R. (2022). EmbedTrack—Simultaneous cell segmentation and tracking through learning offsets and clustering bandwidths. IEEE Access..

[58] Scherr T., Löffler K., Böhland M., Mikut R. (2020). Cell segmentation and tracking using CNN-based distance predictions and a graph-based matching strategy. PLoS ONE.

[59] Zhao M., Jha A., Liu Q., Millis B.A., Mahadevan-Jansen A., Lu L., Landman B.A., Tyska M.J., Huo Y. (2021). Faster Mean-shift: GPU-accelerated clustering for cosine embedding-based cell segmentation and tracking. Med. Image Anal..

[60] Targ S., Almeida D., Lyman K. (2016). Resnet in resnet: Generalizing residual architectures. arXiv.

[61] Xie W., Noble J.A., Zisserman A. (2018). Microscopy cell counting and detection with fully convolutional regression networks. Comput. Methods Biomech. Biomed. Eng. Imaging Vis..

[62] He K., Gkioxari G., Dollár P., Girshick R. Mask r-cnn. Proceedings of the IEEE International Conference on Computer Vision.

[63] Rahmon G., Bunyak F., Seetharaman G., Palaniappan K. Motion U-Net: Multi-cue encoder-decoder network for motion segmentation. Proceedings of the 2020 25th International Conference on Pattern Recognition (ICPR).

[64] Matula P., Maška M., Sorokin D.V., Matula P., Ortiz-de-Solórzano C., Kozubek M. (2015). Cell tracking accuracy measurement based on comparison of acyclic oriented graphs. PLoS ONE.

[65] Ren H., Zhao M., Liu B., Yao R., Liu Q., Ren Z., Wu Z., Gao Z., Yang X., Tang C. (2020). Cellbow: A robust customizable cell segmentation program. Quant. Biol..

[66] Arbelle A., Raviv T.R. Microscopy cell segmentation via convolutional LSTM networks. Proceedings of the 2019 IEEE 16th International Symposium on Biomedical Imaging (ISBI 2019).

